# Long-term fecal diverting device for the prevention of sepsis in case of colorectal anastomotic leakage: an animal experiment

**DOI:** 10.1007/s00384-012-1580-x

**Published:** 2012-09-30

**Authors:** Jae Hwang Kim, Sang Hun Jung, Yong-Jin Kim, Se-ll Park, Dae-Hwan Kim

**Affiliations:** 1 Department of Surgery, School of Medicine, Yeungnam University, Daegu, Korea; 2 Department of Pathology, School of Medicine, Yeungnam University, Daegu, Korea; 3 Department of Veterinary Medicine, Kyungpook National University, Daegu, Korea; 4 Yeungnam University Hospital, Daemyung-Dong Nam-Gu, Daegu, 705-717 Republic of Korea

**Keywords:** Anastomotic leakage, Ileostomy, Low anterior resection, Fecal diversion, Fecal diverting device, Colorectal anastomosis

## Abstract

**Background:**

A new fecal diverting device (FDD) was fabricated for fecal diversion from the proximal colon above the anastomosis to outside the anus for protecting the rectal anastomosis. The aim of the study is to evaluate the safety and effectiveness of the FDD.

**Methods:**

After a pilot study, a prospective observational trial was performed in 34 mongrel dogs. The experiment comprised of segmental resection and anastomosis of the colon, fixation of the FDD, and observation for 3 weeks (*n* = 15) and more than 3 weeks (*n* = 19) without initiation of parenteral nutrition.

**Results:**

Four cases of perioperative death unrelated to the FDD were excluded. Twenty-six (87 %) of the 30 dogs survived. Sixteen (53 %) dogs were able to retain the FDD for more than 3 weeks until 82 days. The autopsy findings revealed that four (15 %) dogs showed colonic wall erosions and mucosal scarring respectively at the band fixation area without evidence of serious septic complications. The surviving dogs retained the FDD for more than 6 days. Mortality occurred in four of the five dogs that expelled the FDD within three postoperative days. A closed abscess cavity as the evidence of anastomotic leakage was noted in seven (23 %) of the surviving dogs.

**Conclusions:**

The newly designed fecal diverting device can be retained for more than 3 weeks until 82 days without any serious complications. The FDD may prevent sepsis in case of anastomotic leakage if it is retained for more than 6 days.

## Background

Anastomotic leakage is the most serious complication of bowel surgery, especially low rectal anastomosis. Various factors contribute to anastomotic leakage. Irving and Goliger thought that residual fecal matter might be a critical cause of anastomotic leakage and that preoperative bowel preparation can reduce the incidence of anastomotic leakage [1]. Recently, there have been doubts whether residual fecal matter can be a major cause of anastomotic leakage [2]. However, most surgeons perform traditional preoperative bowel preparation in patients undergoing colorectal surgery [3].

Defunctioning stoma, a conventional fecal diversion method, is commonly used when anastomotic leakage is a concern. Theoretically, defunctioning stoma prevents catastrophic septic complications by protecting the anastomotic area against further fecal contamination, even if there is anastomotic leakage [4].

Ravo and Ger introduced a technique that could prevent anastomotic complications by diverting the fecal stream from the anastomotic area using a tube device, the Coloshield™ (Deknatel, Inc., Fall River, MA) [5, 6]. Other trials using a condom or a Valtrac™ (biofragmentable anastomosis ring (BAR; United States Surgical, Princeton, NJ))-secured intracolonic bypass (VIB) have reported good results [7, 8]. However, despite successful reports of various devices and methods, there is currently no single device that can be used clinically. The reasons for this could be technical difficulties, lack of control over the duration of device retention inside the bowels, and serious complications, such as bowel perforation. We have designed a new device that diverts the fecal stream similar to that with the Coloshield™, but a different fixation method is used for the application of this new device. Thus, not only can the device be retained inside the bowels as long as required, but it can also be removed anytime. In this animal study, we attempted to evaluate the safety and effectiveness of this experimental fecal diverting device.

## Methods

### Design

This study was designed as a prospective observational trial. A pilot study was performed prior to this study for determination of a stable maintenance schedule for diet and bowel movements while using the experimental device in eight mongrel dogs.

### A newly designed device

**Fig. 1:**
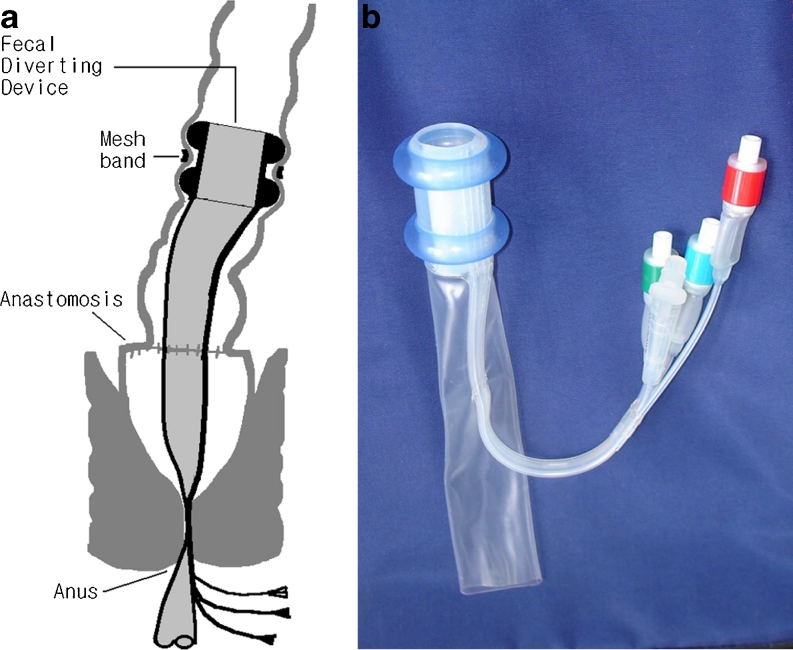
Schematic drawing and photograph of the fecal diverting device

A newly designed device called the fecal diverting device (FDD) is a tubular device made of silicone. There are two tire-like dumbbell-shaped outer balloons on the head portion of the device which help to fix the device on the colon proximal to the anastomotic area without sutures. A nonabsorbable polyester mesh (Parietex®; Sofradim Co., Trevoux, France) band was used to fix the head portion of the device externally on the colon (Fig. 1). Inside the head portion of the device, there is an inner balloon, which permits or blocks the flow of the bowel contents. The tail, i.e., the thin tube below the head portion, is long enough to hang out from the anus. Fecal matter enters from the proximal colon into the head portion of the device and exits from the anus through the tail portion of the device, thereby avoiding fecal contamination of the anastomosis.

### Animal experimental procedures

Thirty-four healthy mongrel dogs (males 12, 15–27 kg, median weight 19 kg) were used in the study. Dogs were kept in the cages where they would be placed after surgery for at least 5 days for acclimatization to the environment prior to surgery. There was no preoperative fasting or bowel preparation. However, second-generation cephalosporin was injected 30 min prior to surgery.

The operation was performed using a low midline incision under intravenous ketamine anesthesia. After laparotomy, intraoperative colonic lavage was performed through the anus using the New Intraoperative Colonic Irrigator (NICI®, MITech Co., Seoul, Korea) [9] in order to achieve easy manipulation of the colon. For irrigation, approximately 2,000 cm^3^ of warm tepid water was used; the washout procedure took 5 min. We designed an ischemic anastomotic model for easier anastomotic disruption in order to be able to assess the efficacy of the FDD more accurately.

Colonic resection and anastomosis procedure was performed at approximately 10 cm from the anus, around the inferior mesenteric artery. An avascular colonic segment measuring approximately 5 cm was created by devascularization of the pericolic mesenteric vessels and inferior mesenteric artery for inducing ischemia at the anastomotic area. A circular stapler (Ethicon Endo-Surgery, Johnson & Johnson, USA) was introduced through the anus and advanced up to the devascularized segment. On this ischemic colonic segment, a 5–0 silk tie was placed between the anvil and the stapler body for resection and anastomosis. Approximately 2 cm of the colonic segment was resected using this stapling procedure.

**Fig. 2:**
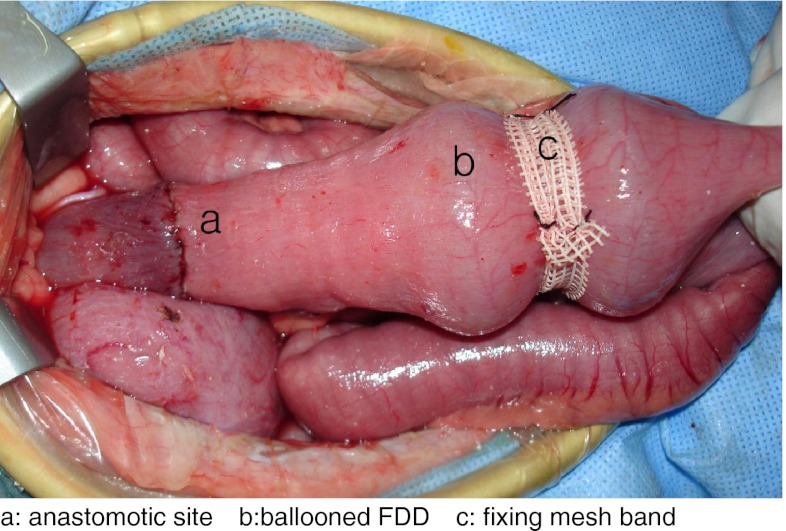
The FDD (fecal diverting device) is fixed by a mesh band at 5–10 cm proximal to the anastomotic site

After stapling, the head portion of the FDD was inserted into the anus with the inner balloon inflated and the outer balloons deflated. The FDD was passed through the anastomotic ring and was placed in the colon at approximately 5–10 cm proximal to the anastomotic ring. In order to fix the device, first, a mesh band was placed externally between the two outer balloons. Afterwards, the two outer balloons were inflated into a dumbbell shape so that the FDD would not move away from the site. Several silk 3–0 stitches were applied for fixing the mesh band on the colon in order to prevent the movement of the mesh band (Fig. 2). The tail portion of the device that was hanging outside the anus was severed at the anal level in order to protect the device from damage due to external factors or by the dogs themselves. After the surgical procedure, dogs were kept in cages for a duration of 3 weeks (*n* = 15) and more than 3 weeks (*n* = 19). Water and dog food were kept in the cages postoperatively. In order to ease the flow of the fecal matter through the device after surgery, lactulose (2 cm^3^/kg/day) and polyethylene glycol solution (50 cm^3^/kg/day) were thoroughly mixed in food and water, either individually or in combination. Initially, 15 dogs were kept under observation for a period of 3 weeks. Then, 19 dogs were kept under observation for as long as possible, without any set timeframe. The dogs were monitored for unexpected events throughout the experimental period over closed-circuit television (CCTV). Dogs were killed by administration of excessive doses of ketamine for evaluating the intraabdominal findings.

If the device was expelled before 3 weeks, the observation period was 5–7 days longer in order to examine the follow-up effect of early expulsion of the FDD. We removed the FDD in 15 dogs if it was retained until 3 weeks. We experienced successful removal of the FDD without anesthesia in the pilot study by pulling the tail portion (composed of a thin silicone tube and catheter) of the FDD after deflating both the outer balloons.

### Objectives

The primary end points of the study were identification of possible bowel wall injury at the FDD fixation area and duration of FDD retention inside the bowels. The secondary end point was anastomotic complications. In addition, intraabdominal findings and wound complications were also evaluated.

Gross findings on examination were intraperitoneal adhesions, bowel injury, inflammation around the mesh area or anastomotic area, and any other unexpected findings. The segment including the mesh band and anastomosis was resected and evaluated macroscopically and microscopically for identification of the various mucosal and bowel wall changes. After macroscopic examination, representative sections were taken from the colon for light microscopic examination. Tissues were fixed in 10 % formalin and embedded in paraffin. Four-micrometer sections were stained with hematoxylin and eosin (H&E).

Expelled FDD, CCTV findings, and mesh band status were analyzed for evaluation of the causes of premature FDD expulsion. In this experiment, anastomotic leakage was defined as visible disruption of anastomosis, secondary peritonitis, and presence of intraperitoneal gas or fecal soilage. Evidence of anastomotic leakage was defined as the presence of a closed abscess cavity, mucosal or muscular discontinuation in the anastomotic area.

In case of premature death before 3 weeks, an autopsy was performed within 24 h after death. The dogs that died during surgery or those that died due to any cause other than problems caused by the FDD and mesh band were excluded from the study.

### Statistical analysis

We measured the mortality incidence rate, bowel injury rate, duration of FDD retention inside the bowels for more than 3 weeks, anastomotic leakage rate, and the 95 % confidence interval (CI) for each rate. These data were obtained using statistical package SPSS version 19.0.

## Results

**Fig. 3:**
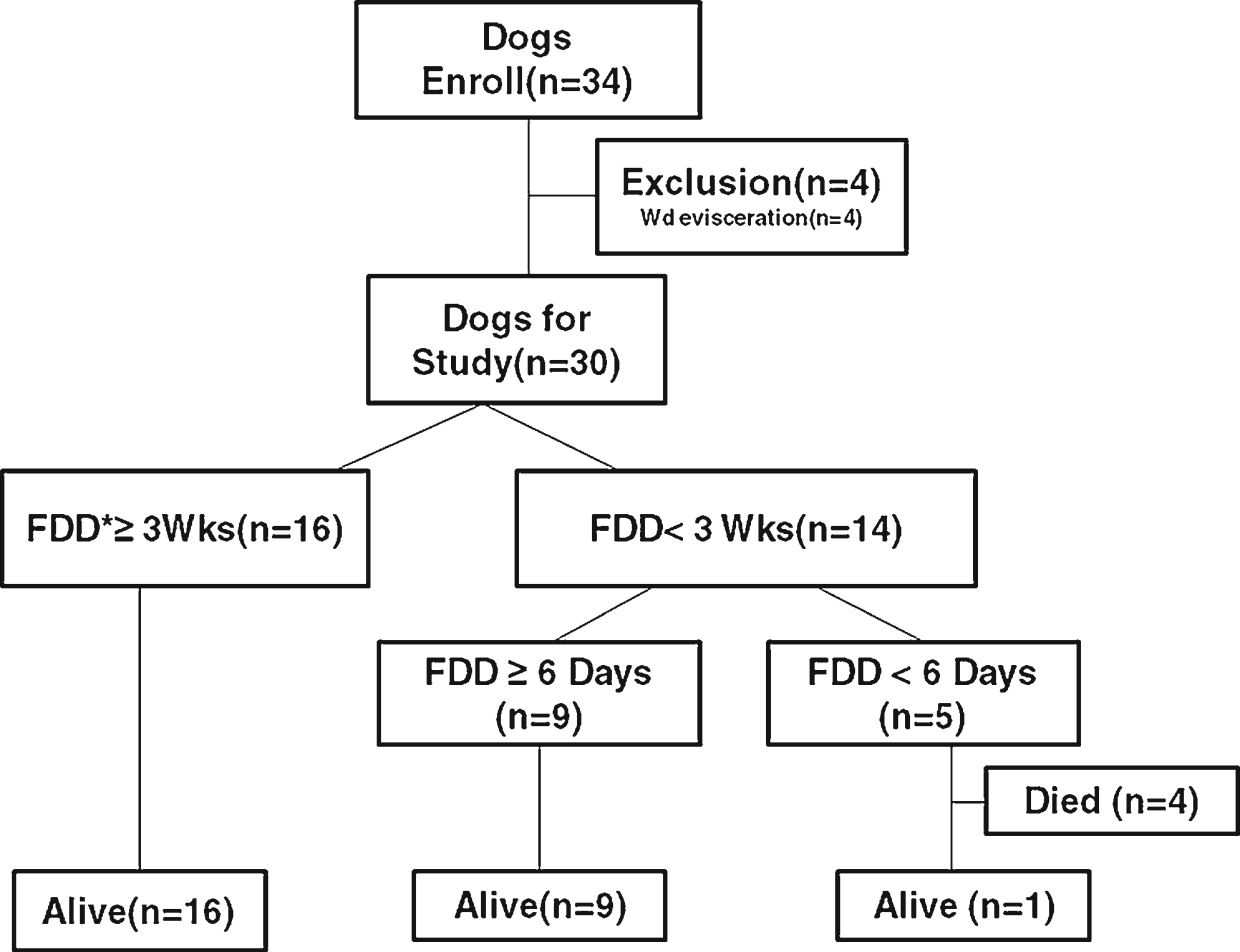
Flow chart of the dogs included in this study

A total of 34 dogs were enrolled in this study. The reasons for exclusion from the study, the duration of FDD retention, and FDD failure are presented in Fig. 3.

### Exclusion

Four of the 34 dogs were excluded from this study due to the occurrence of major wound evisceration within 5–8 postoperative days. Two dogs were found dead, and the possible cause of death was hypovolemic shock due to massive hemorrhage from multiple injured visceral vessels. Another two dogs were found alive, however, with herniated and injured multiple visceral organs. They were euthanized with a high dose of ketamine. We think that the cause of wound evisceration was excessive straining in order to push the foreign body out from the anorectum, and this was frequently noted in the CCTV records. In autopsy findings, the FDD, FDD fixation area, and anastomotic area were intact. We included the remaining 30 dogs in this study.

### Survival and duration of the FDD retention inside the bowels

Twenty-six (87 %; 95 % CI, 62.3–96.2 %) of the 30 dogs survived. In 16 of the 30 dogs (53 %; 95 % CI, 34.3–71.7 %), the device was retained inside the bowels for more than 3 weeks without mortality. The longest time period for which the device was retained inside the bowels was 82 days.

Among the 14 dogs (47 %; 95 % CI, 28.3–65.6 %) in whom the device was expelled within less than 3 weeks, 4 dogs (13 %, 95 % CI, 3.7–30.7 %) died within 6 postoperative days after expulsion of the device within 3 postoperative days. The causes of expulsion of the device were considered to be FDD prototype manufacturing errors and low mesh band tension (Table 1). Autopsy findings showed the accumulation of purulent ascitic fluid and fecal matter with a foul odor in the abdominal cavity. Partial anastomotic disruption was clearly noted, and it was considered to be the cause of generalized peritonitis. In the remaining ten dogs that survived, the device was expelled after 6–19 postoperative days in nine dogs and after 2 postoperative days in the remaining one dog. None of the dogs in whom the device was retained inside the bowels for more than 6 days died.

**Table 1:** A significant association between FDD expulsion and mortality was noted

Dog^a^	Cause of FDD out	Wt (kg)	FDD out (POD)	Die (POD)
8	Device failure^b^	23	2	3
9	Device failure	23	3	6
10	Loose band	27	1	4
27	Device failure	20	1	5
29	Device failure	20	2	Live

The causes of FDD expulsion, the date of FDD expulsion, and death are listed

*FDD* fecal diverting device, *Wt* weight of dogs, *POD* postoperative day

^a^Serial number of experimental dog

^b^Flattening or tearing of one of FDD outer balloons

### Bowel wall changes in the FDD fixation area of the resected bowel segment

**Fig. 4:**
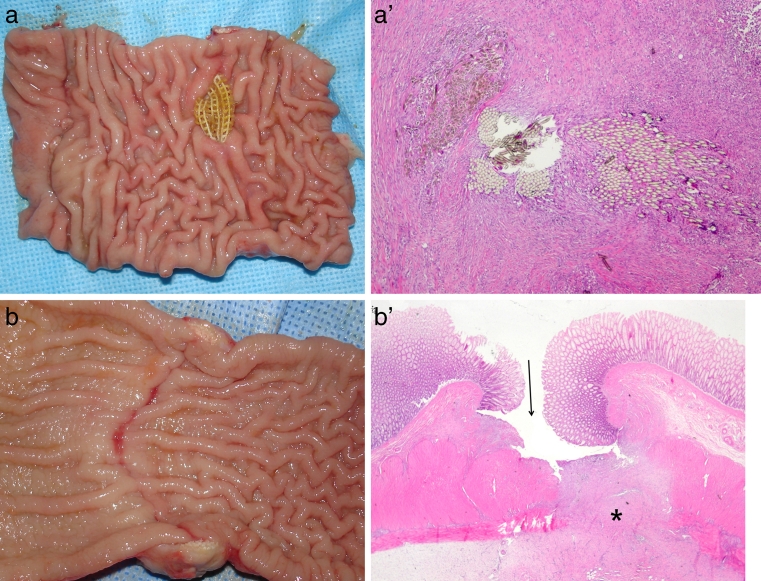
Bowel wall erosion (**a**) and linear mucosal scarring (**b**) developed at the FDD (fecal diverting device) fixation area. **a** Macroscopic findings of the sectioned colonic segment containing the band fixation area and anastomotic ring; partially penetrated mesh band material is noted on the mucosal surface. **a**’ Microscopic findings of the sectioned bowel wall containing the mesh band material during the process of erosion; Cross sections of the bundles of mesh bands are noted in the submucosa. They are surrounded by dense fibrosis rather than acute inflammation. H&E, ×100. **b** Macroscopic findings of the sectioned bowel lumen at the band fixation area; A transverse linear scar is noted along the placement of mesh band. **b**’ Microscopic findings of the sectioned bowel wall at the band fixation area; Surface mucosa is ulcerated (*arrow*). Underlying submucosa and muscular layer are replaced by fibrosis (*asterisk*). H&E, ×40

Bowel wall erosion was noted in 4 of the 26 dogs that survived (15 %; 95 % CI, 4.3–34.8 %). Linear mucosal scars were noted in four dogs (15 %; 95 % CI, 4.3–34.8 %; Table 2). Bowel wall changes appeared to be aggravated with an increase in the duration of FDD fixation. However, despite the development of bowel wall changes, there was no gross evidence of local sepsis around the erosion or the mucosal defect site. Microscopic findings revealed that the erosion caused only a minimal to mild inflammatory response, rather than severe inflammation. Loss of mucosa and fibrotic tissue changes in the muscle layer were noted at the site of erosion and mucosal scarring of the bowel wall (Fig. 4a, a’, b, b’).

**Table 2:** Bowel wall changes were identified at the FDD fixation area

Dog^a^	Wt (kg)	FDD out (POD)	Bowel wall changes
6	15	3 weeks	Encircling linear mucosal defect
12	15	3 weeks	Encircling linear mucosal defect
14	20	3 weeks	Small mucosal defect
16	20	70	Encircling linear mucosal defect
17	23	82	Erosion 2.5 cm in length
18	22	43	Erosion 1 cm in length
23	18	9	Erosion 1 cm in length
28	20	22	Erosion 2 cm in length

Bowel wall changes were aggravated with an increase in the duration of FDD retention

*FDD* fecal diverting device, *Wt* weight of dogs, *POD* postoperative day

^a^Serial number of experimental dog

### Findings in the anastomotic area showed evidence of anastomotic leakage

Continuous bowel wall defects with a closed abscess cavity were observed in seven dogs (23 %; 95 % CI, 9.9–42.3 %), and mucosal discontinuation was observed in three dogs (10 %; 95 % CI, 2.7–28.1 %). Closed abscess cavities were found in the dogs in which the FDD was retained inside the bowels for 7–21 postoperative days (Table 3). Most of the dogs who had a closed abscess cavity or mucosal disruption showed symptoms of postoperative ileus such as poor oral intake and decreased general activity for several days. Microscopic examination revealed evidence of a severe inflammatory process, such as cluster of inflammatory cells, foreign body granuloma, and severe extraluminal fibrosis of the closed abscess cavity.

**Table 3:** Findings in the anastomotic area of dogs with evidence of anastomotic leakage

Dog^a^	Wt (kg)	FDD out (POD)	Anastomotic area	Associated findings
6	15	3 weeks	Wall disruption	Closed cavity^b^
11	24	3 weeks	Mucosal disruption	Adhesion^c^
12	15	3 weeks	Wall disruption	Closed cavity
14	20	3 weeks	Wall disruption	Closed cavity
17	23	82	Mucosal disruption	Adhesion
21	17	19	Wall disruption	Closed cavity
23	18	9	Wall disruption	Closed cavity
24	20	21	Wall disruption	Closed cavity
30	22	7	Wall disruption	Closed cavity
34	20	6	Mucosal disruption	Adhesion

*FDD* fecal diverting device, *Wt* weight of dogs, *POD* postoperative day

^a^Serial number of experimental dog

^b^Healed closed abscess cavity

^c^Adhesion with adjacent organ of structures

### Autopsy findings in the dogs that survived

On gross examination, mostly minimal to moderate adhesions were observed around the band fixation area and anastomotic area. Most of the times, no difficulties were encountered during the dissection of these adhesions. However, in ten dogs with evidence of anastomotic leakage, moderate to severe adhesions were observed around the anastomotic area.

### Removal of the FDD

In the 15 dogs kept under observation for 3 weeks, the FDD was expelled spontaneously in 10 dogs. In the remaining five dogs, the FDD was removed manually after it was retained until 3 weeks. Even though there was minimal to mild resistance while pulling the FDD out, we successfully explantated the FDD in all the dogs. And there were no evidence of intraabdominal injuries around the FDD fixation area or anastomotic area in the autopsy findings after manual removal of the FDD. In the remaining 19 dogs, spontaneous expulsion of the device occurred before or after 3 weeks.

### Other findings

The causes of early expulsion of the device were evaluated. Device failure occurred in 7 (50 %) of 14 dogs. One or both the outer balloons collapsed due to tearing or incomplete sealing of this prototype balloon device. Early device expulsion occurred in five (36 %) of seven remaining dogs as a result of the body portion of the FDD or catheters for outer balloons being bitten off by the dogs. In the remaining two dogs, the device was expelled easily as a result of a loose mesh band (Table 4.). Excessive straining in order to expel the device was noted in almost all dogs that were identified in CCTV recordings. Wound infection and partial wound disruption occurred in seven dogs (23 %) and two dogs (6 %), respectively.

**Table 4:** Possible causes of premature FDD expulsion

Cause of FDD out	No. of dogs	FDD out (POD)
Device failure^a^	7	1^b^, 2^b^, 2, 3^b^, 6, 7, 19
Damage by dog bite	5	6, 6, 9, 11, 11
Loose mesh band	2	1^b^, 9

*No.* number, *POD* postoperative day

^a^Flattening or tearing of one of FDD outer balloons

^b^Died

## Discussion

As reported in the literature, the first attempt towards prevention of anastomotic leakage by fecal diversion was performed by Lanfrank in 1565 [10]. In 1984, Rabo and Ger introduced a unique method for preventing fecal contamination of the anastomotic area by diverting the fecal matter through a silicone tube. This device was named Coloshield™, and it showed successful clinical results [11–18]. The same method with the use of a condom rather than the Coloshield™ also reported successful results [7, 19]. The VIB was applied in the animal experiments and clinical study with successful results [8, 20]. However, despite these successes, currently, there is no single device which is routinely used clinically.

The shortcomings of these various methods in which unique devices are used are that they are technically demanding, are associated with a risk for bowel perforation, and there is lack of control over the duration of device retention inside the bowels. In the case of Coloshield™ and condom, continuous suturing of only the mucosa and muscularis mucosa layer is recommended. Egozi et al. [21] reported a case of peritonitis caused by the use of Coloshield™ which led to erosion and perforation of the colonic wall because of a comparably solid tube material. This phenomenon has already been described in the Ross’s report of an animal experiment [22]. Lack of control over the duration of the device retention inside the bowels is a major problem associated with the use of these alternatives. Coloshield™ is expelled naturally when necrosis of the sutured mucosal and submucosal tissue occurs. The duration of the Valtrac™ ring inside the colon is also limited.

The density of collagen, which is the most crucial for healing of anastomosis, is the highest at 1 week after anastomosis [23]. The time duration of retention of Coloshield™ is 1–3 weeks and that for Valtrac-secured intracolonic bypass is usually 2 weeks. Therefore, in most cases, theoretically it allows safe anastomosis. However, if partial dehiscence occurs due to ischemic tissue necrosis, the anastomotic healing period can be prolonged. In various situations, the time duration required for a safe anastomosis might be prolonged compared to that in a normal healthy intestine. In the case of preoperative radiation therapy or chronic use of steroids, a period of 1–2 weeks cannot be considered sufficient for wound healing [24].

The reason for setting the time duration for device retention inside the bowels in this study was based on the fact that the majority of anastomotic leaks occur within 1–2 weeks. We thought that the 3-week period of fecal diversion was sufficient for anastomotic wound healing provided there were no other factors which could inhibit wound healing. As a result, in 53 % of dogs, the device was successfully retained inside the bowels for more than 3 weeks, and the longest period of device retention was 82 days. This result proves that the duration of fecal diversion can be controlled for extended periods using the FDD if the device is designed using high standards. Thus, it is thought that the FDD might function like an abdominal stoma in the patients, even in those who require an extended period of fecal diversion.

We did not evaluate the adverse effects of the FDD on the anal sphincter complex in this study; however, if we use the FDD in a clinical setting then this might be an issue that needs to be addressed. Similar examples found in the literature are the application of continent anal plug and Zassi Bowel Management System (Zassi Medical Evolutions, Fernandina Beach, Florida) [25, 26]. It has been reported that there was no sphincter injury at a maximum duration of retention of 37 and 59 days.

In a clinical setting, if there is postoperative anastomotic leakage, preceding stoma creation or Hartmann’s procedure is performed conventionally. Even though these are well-known techniques, another operation is needed in the near future for reducing the stoma. Furthermore, patients could experience various stoma-related complications before and after reduction of the stoma. The situation in our study is not similar to these situations of postoperative anastomotic leakage without preceding stoma creation; however, we can consider application of the FDD as an alternative procedure to defunctioning stoma or Hartmann’s operation. The benefits of application of the FDD could be avoiding stoma-related complications and another operation in the near future. However, we cannot be sure of the safety of the FDD because of the possible occurrence of unknown complications due to the use of the FDD. The most critical complication is the development of anastomotic stricture after leakage. Subsequent local sepsis would be more serious in cases without a stoma than in those with a stoma. In the current study, there was no case of failure of explantation of the device even though there was development of strictures after leakage. However, we need to perform further studies concerning this issue to confirm the safety of the FDD.

The most critical issue in this study is the safety of the fixation method of the FDD. It is well known that the bowel is very sensitive to compression. Possible injury to the band fixation area is the most important concern if the duration of device retention inside the bowels is prolonged. Erosion occurs following adjustable gastric banding procedures performed for morbid obesity or artificial anal sphincter procedures performed for fecal incontinence. As has been reported, the frequency of bowel wall erosion, as a long-term complication, also increases with time; in particular, the frequency of bowel wall erosion increases with the use of an artificial anal sphincter that compresses the bowel wall more than the adjustable gastric banding [27, 28]. In our study, the erosion supposedly resulted from continuous pressure between the band and the solid head portion of the device. If the band is kept too loose so as to decrease the pressure, the device would be expelled easily. On the other hand, if the band is kept too tight so as to hold the device securely, the pressure rises inevitably. We applied a slightly longer mesh band than the circumference of the bowel retaining the FDD. It is very encouraging to note that the fixation of the device with the application of tension resulted only in several erosions and mild mucosal scars, and did not lead to any critical complications or mortality. We used a nonabsorbable mesh band in this study because we could not find an ideal absorbable mesh material. It is also very encouraging to note that 85 % of the dogs that survived were erosion-free. We think that the problem of erosion can be solved if we use an ideal absorbable mesh material with an appropriate shape and tension in the future. We think that the use of absorbable mesh materials could reduce the long-term problem of erosion even if erosion develops.

Successful removal of the FDD was observed in the pilot study even though there were some difficulties because of poor cooperation of the dogs. The difficulties faced during the FDD removal in this study were not only the animal’s poor cooperation but also minimal to mild resistance while pulling the FDD out. We think that the FDD removal could be painful for the patient if it would be applied clinically although the explantation procedure might be successful. This study has a limitation regarding the safety with respect to the manual removal of the FDD because of the small sample size. However, the fact that there was no evidence of intraabdominal injuries around the FDD fixation area or anastomotic area in autopsy findings after manual removal of the FDD indicates that serious complications may not occur.

We observed early expulsion of the device in 14 dogs in this study. Early expulsion of the device not only results in loss of function but also in possible damage to the anastomosis as the device descends and passes through the anastomotic area. Four of 14 dogs with early device expulsion died of generalized peritonitis due to anastomotic breakdown. Expulsion of the FDD occurred within three postoperative days in these four dogs. However, expulsion of the FDD after 6 postoperative days did not result in death. This result was similar to the results reported by Ravo or Ye which show that in most cases, anastomosis was protected by fecal diversion for more than a week. We can assume that protection of the anastomosis for more than 6 days is sufficient in this dog model.

The most concerning anastomotic problem occurs during extraperitoneal rectal anastomosis in colorectal surgery. We did not use the distal rectum in the study because anastomosis near the anus can be damaged in dogs due to straining and biting. We tried to make the anastomotic complications in the colon easier for the substitution of low rectal anastomosis. The experimental animal model developed for this study had an inadequate blood supply at the anastomotic site which can result in a high anastomotic complication rate, and the efficacy of the FDD was assessed against this background. According to the results, evidence of anastomotic leakage based on autopsy findings was noted in seven dogs with a closed abscess cavity, in three dogs with mucosal discontinuation, and in four dogs with generalized peritonitis due to anastomotic leakage. We think that in this study, 46 % of anastomotic breakdown developed due to the use of this experimental model which provided an excellent background.

Regarding early expulsion of the device, we think that the problem involving experimental animals is in itself not a big one; however, we can understand that there might be some discomfort to the experimental animals because of the procedure. The problem regarding manufacturing of safer devices and applying appropriate band tension can be solved, and it is a subject for future trials.

In conclusion, to the best of our knowledge, the technique of retaining an intraluminal solid device with an extraluminal band at the site of a hollow viscus has not been reported in the literature until now. In this study, the newly designed fecal diverting device (FDD) was retained inside the bowels for more than 3 weeks, and it effectively protected the anastomosis against fecal contamination by diverting the fecal stream. Erosion developed in 15 % of cases; however, there were no cases of septic or critical complications. The longest duration of device retention was 82 days. In this animal study, despite the occurrence of anastomotic leakage, the FDD was able to prevent a catastrophic cascade if it was retained inside the bowels for more than 6 days. Further studies with the use of an absorbable mesh band and reliable device are needed in order to verify the safety of the FDD before its clinical application.
